# Efficient Transovarial Transmission of *Babesia* Spp. in *Rhipicephalus microplus* Ticks Fed on Water Buffalo (*Bubalus bubalis*)

**DOI:** 10.3390/pathogens9040280

**Published:** 2020-04-11

**Authors:** Dasiel Obregón, Belkis Corona-González, Adrian Alberto Díaz-Sánchez, Yasmani Armas, Eugenio Roque, Márcia Cristina de Sena Oliveira, Alejandro Cabezas-Cruz

**Affiliations:** 1 School of Environmental Sciences, University of Guelph, Guelph, ON N1G 2W1, Canada; 2 Center for Nuclear Energy in Agriculture, University of São Paulo, Piracicaba, São Paulo 13400-970, Brazil; 3 Centro Nacional de Sanidad Agropecuaria (CENSA), San José de Las Lajas, Mayabeque 32700, Cuba; bcorona@censa.edu.cu (B.C.-G.); adiasanz88@gmail.com (A.A.D.-S.); 4 Department of Biology, University of Saskatchewan, Saskatoon, SK S7N 5A2, Canada; 5 Faculty of Veterinary Medicine, Universidad Agraria de La Habana, San José de Las Lajas, Mayabeque 32700, Cuba; yarmas2012@gmail.com (Y.A.); roque@unah.edu.cu (E.R.); 6 Embrapa Pecuária Sudeste, São Carlos, São Paulo 13560-970, Brazil; marcia.sena-oliveira@embrapa.br; 7 UMR BIPAR, INRAE, ANSES, Ecole Nationale Vétérinaire d’Alfort, Université Paris-Est, 94700 Maisons-Alfort, France

**Keywords:** water buffalo, cattle, ticks, reproductive efficiency, *Babesia*, qPCR

## Abstract

Water buffaloes can be infected by tick-borne pathogens (TBPs) in endemic areas where cattle and buffalo coexist. Among TBPs affecting buffaloes is the Apicomplexan hemoparasites *Babesia bovis* and *B. bigemina*, transmitted by *Rhipicephalus microplus* ticks. However, little empirical evidence exists on whether buffalo can support TBPs’ infection and transmission. A cohort study was designed to measure the infestation levels of *R. microplus* in buffaloes as well as the ability of buffalo-fed ticks to transmit *B. bovis* and *B. bigemina* to their offspring. Tick infestation of different life stages was quantified in cattle and buffalo kept in field conditions in western Cuba. Engorged adult female ticks were allowed to lay eggs in controlled conditions of humidity and temperature, and reproductive parameters were measured and analyzed. Hosts and tick larvae were tested for the presence of *Babesia* spp. using species-specific qPCR assays. Tick infestation was not observed in adult buffaloes. However, buffalo and cattle calves were equally infested, although the larval survival rate was higher in cattle calves than in buffalo calves. All larval pools (31) obtained from the adult female ticks were positive for *B. bovis,* whereas only 68% (21/31) was positive for *B. bigemina*. Among the 10 larval pools negative for *B. bigemina*, three proceeded from adult females fed on *Babesia*-negative buffaloes. The other seven pools were from *Babesia*-positive animals, three from cattle and four from buffalo calves. *Babesia* infection levels in tick larvae, quantified by qPCR, were similar in female ticks fed on buffalo and bovine calves. We conclude that water buffalo can sustain tick vector populations and support *Babesia* infection in levels high enough as to be infective for ticks. Our results also validated the hypothesis that adult female ticks fed on buffalo can transmit the pathogens *B. bovis* and *B. bigemina* to their offspring. Nevertheless, further laboratory studies are needed to address the question of whether the transovarial transmission of *Babesia* occurs in the following settings: (1) When adult females are infected previous to the feeding on the buffalo or/and (2) when the adult females acquire the infection while feeding on the buffalo.

## 1. Introduction

Bovine babesiosis, caused by the protozoan parasites *Babesia bovis*, *B. bigemina*, and *B. divergens* (Apicomplexa: Babesiidae), is a tick-borne disease (TBD) posing a constraint for livestock production in tropical and subtropical regions of the world. Specifically, *B. divergens* has been reported only in northeast Europe [1,2], whereas *B. bovis* and *B. bigemina* are widespread in several countries in Latin America, Africa, Australia, and Asia. The one-host ixodid tick *Rhipicephalus microplus* is the vector of these pathogens in the Caribbean and South American countries [3,4]. These piroplasms replicate within tick tissues and have transstadial and transovarial transmission [5,6]. This adaptation ensures the long-lasting persistence of infection in endemic areas, since ticks remain infected and infective for several generations without the need of reinfection [6]. In the vertebrate hosts, *Babesia* spp. infect the erythrocytes, producing a disease characterized for hemolytic anemia. Bovines (cattle) are the maintenance hosts of *B. bovis* and *B. bigemina*. Other ruminants including *Odocoileus virginianus* (white-tailed deer) [7], *Syncerus caffer* (African buffalo) [8], and *Bubalus bubalis* (water buffalo) [9] can be carriers of these pathogens. However, whether carrier animals can act as a reservoir of these pathogens remains to be established [9].

*Rhipicephalus microplus* is the most important tick infesting cattle and represents 57% of the ixodid ticks’ population in Cuba [10,11]. The climatic conditions that prevail in Cuba allow the development of tick life cycle on grassland during all months of the year [12,13]. Buffaloes are also parasitized by *R. microplus* in Cuba, although with a low infestation load [14]. Benitez et al. [15] showed experimentally that *R. microplus* can complete its life cycle in buffaloes. These authors also observed that only 5% of the tick larvae that fed on buffaloes completed their life cycle, while in cattle, 12% of the larvae completed the cycle. The elimination of ticks at different life stages can influence the dynamics of host infection by these piroplasms since only larvae transmit *B. bovis* while *B. bigemina* is transmitted by nymphs and adults [5].

Buffalo herds are increasing in Cuba and often coexist with cattle herds [16]. These animals may be carriers of *Babesia* spp. [9,17,18]. Therefore, they are potential reservoirs for these pathogens and can affect the eco-epidemiology of babesiosis in cattle [19,20,21,22]. However, evidence of the vectorial capacity of *R. microplus* that feed on carrier buffalo is scarce. As far as we know, no study has measured the transovarial transmission of *Babesia* spp. on ticks that feed on buffaloes. Thus, in this study, we verified the levels of *R. microplus* infestation in calves and adult buffaloes and compared to cattle, when raised together or in distant farms. We also quantified the levels of infection by *B. bovis* and *B. bigemina* in the progeny of ticks collected on these animals.

## 2. Results and Discussion

In this work, no tick infestation was found in the adult buffaloes of either the neighboring or control herds (Table 1). In contrast, buffalo calves were infested in both groups, with prevalence similar to that of adult cattle and cattle calves (*F* = 1.52, *p* > 0.05). The highest parasitic load among all the groups was observed in buffalo calves in immediate proximity to cattle herds (*F* = 2.49, *p* = 0.04) (Table 1). Similar results were obtained in Thailand, where Nithikathkul et al. [23] observed that adult buffalos were less infested by *R. microplus* than cattle in endemic areas. In addition, a previous study in the same region in Cuba reported that, unlike adult buffalo, buffalo calves were susceptible to tick infestation, presumably because they had thinner and hairier skin [14]. This result suggests a natural resistance of adult buffaloes to ticks. The resistance of adult buffaloes to tick infestation seems to be acquired since calves are not resistant to this ectoparasite. Another factor to consider may be that buffalo calves usually have less access to wallows (water and mud) than adult buffalo in the study region, which constitutes a biological control for ticks [15,19].

The nymphs/larvae ratio was higher in bovine calves when compared to buffalo calves (*F* = 40.9, *p* < 0.05, Table 1). These results are consistent with a study conducted in field conditions in Brazil in which the proportion of nymphs (21.3%) observed in buffaloes was much lower than that of larvae (74.7%). In that work, they also observed that larvae attack in buffalo calves happened mainly during the firsts months of life, usually causing intense inflammatory reactions at the skin sites more bitten by ticks [24,25]. Furthermore, our results are comparable with the findings of Benitez et al. [15], who carried out an experimental infestation of cattle and buffalo calves in Argentina using 10,000 *R. microplus* larvae and observed that the larval survival was lower in buffalo than in cattle [15]. These authors also observed that only 5.4 % and 12% of larvae survived until the adult stages in buffalo and cattle, respectively. Benitez et al. [15] also described an intense inflammatory response on buffalo at skin sites where a large number of larvae were attached. The inflammatory reaction was not observed in cattle [15], suggesting that the immune system of buffalo may be more reactive to tick saliva components than that of cattle. Accordingly, in this work, inflammatory reactions were observed in the skin of buffaloes infested by ticks (Figure S2), but were not observed in cattle. These results suggest that buffalo acquire immune resistance to tick infestation, whereas cattle remain tolerant or susceptible to ticks.

The immunological mechanisms underlying the natural resistance of buffalo to tick infestations remain to be identified. In cattle, host genetic factors define the immune resistance to ticks [26], and tick resistance is an inherited trait in *Bos taurus indicus* that can be passed to *B. t. taurus* by crossbreeding of these two subspecies [27]. The precise mechanisms underlying tick resistance in cattle remains poorly understood, but several host genetic factors associated with tick resistance and susceptibility have been identified [26,28]. Despite a combination of humoral and cellular immune response mechanisms seeming to be involved [26], the evidence for a correlation between the level of tick infestation and the level of immunoglobulin G (IgG) to tick antigens is contradictory [26]. However, there is consensus in that different subsets of inflammatory cells play an important role in tick resistance in bovines [26,29,30]. For example, higher numbers of immune cells involved in the histamine response of the skin, eosinophils, mast cells, and basophils, were found in the skin of resistant cattle [26,28].

The weight of fully engorged adult females that fed on buffalo calves (0.150 ± 0.028 g) was similar to that of ticks that fed on cattle (0.151 ± 0.033 g). Similar results were reported by Benitez et al. [15], who found no differences in the body weight of adult female ticks that fed on cattle and buffaloes, or in the times of preoviposition, oviposition, and incubation. The reproductive performance of adult female ticks is shown in Table 2. No significant difference was found in the reproductive parameters CE (*F* = 0.93, *p* = 0.47), HR (*F* = 0. 34, *p* = 0.88), and REI (*F* = 0.77; *p* = 0.57) of adult females fed on bovine or buffalo calves. After observing similar results, Benitez et al. [15] concluded that *R. microplus* adult females can complete their life cycles in buffalo calves and cattle with comparable reproductive efficiency.

The qPCR analysis revealed that the 31 groups of tick larvae (Figure S3) and the 31 hosts tested were positive for *B. bovis*. However, only 68% (21/31) of the larval groups was positive for *B. bigemina*. Of the 10 larval groups negative for *B. bigemina*, three were from qPCR-negative buffalo calves, and the other seven were from adult females fed on qPCR-positive animals (four buffalo calves and three adult cattle). The infection levels of *B. bovis* were similar in larvae from females fed on buffalo and bovine calves (Table 3), while larvae from adult cattle harbor the highest values of infection (*F* = 4.90, *p* = 0. 05). In the case of *B. bigemina* there were no differences in the infection levels in tick larvae relative to host type (*F* = 3.06, *p* = 0.06). Besides, we found higher values in the count of parasitic infection by *B. bovis* than *B. bigemina* (*t* = 7.08, *p* < 0.05).

The higher frequency of *B. bovis* compared to *B. bigemina* in the offspring of adult females of *R. microplus* fed on cattle is a distinctive epidemiological feature of our study, since most studies report that *B. bigemina* infection is more frequent than *B. bovis* in ticks fed on cattle [5,31]. Specifically, research conducted in Australia revealed that only ~0.04% of larval ticks were infected with *B. bovis* on farms with *B. t. taurus* cattle, whereas the infection with *B. bigemina* was usually higher (i.e., ~0.23%) [32]. As a consequence, a higher transmission rate of *B. bigemina* than of *B. bovis* could be expected in regions where both *Babesia* species are present, therefore, causing the highest prevalence of *B. bigemina* in cattle herds [5,33]. However, our results are consistent with those of an epidemiological study previously conducted in the same region as our study, in which a high molecular prevalence of both *B. bovis* and *B. bigemina* was found in buffalo [22]. Eco-epidemiological factors favoring the presence of *B. bovis* in this region of Cuba should be studied, as *B. bovis* could cause severe disease in susceptible cattle, particularly in South America and Australia [5,34].

A limitation in this work was that the qPCR quantification of *Babesia* spp. was carried out on tick larval pools (~100 larvae from egg batches laid by 10 engorged females). This approach could have influenced the high frequency of positive samples detected in our study, thus overestimating the level of transovarial transmission of *Babesia* spp., despite low transovarial transmissibility of these apicomplexan parasites [35]. Future studies including water buffaloes should quantify the infection level in each larva and establish the proportion of larvae that acquired the *Babesia* transovarially. This aspect is essential to determine the likelihood of host exposure to these hemoparasites when buffaloes are present in endemic areas with a potential impact on the epidemiology of bovine babesiosis (e.g., water buffalo could have a dilution effect [36] on the dynamics of *Babesia* spp. transmission). In this regard, studies carried out in Australia compared the transmission rates of *B. bovis* in *B. t. taurus* and *B. t. indicus* and concluded that the breeding of tick-resistant cattle might lead to the disappearance of ticks in environments not suitable for tick survival [33]. This situation could lead to low infection rates and lack of immunity on cattle herds, therefore, posing a threat to endemic stability [5,33].

In this work, the molecular quantification of the pathogens in vertebrate hosts revealed that the infection levels of *B. bovis* were higher in cattle than in buffalo when both species coexisted (neighboring-herds) (*F* = 7.98, *p* < 0.05), however, the infection levels were similar to those observed in the control groups. No differences were observed between age groups from the same host species (Table 4). In the case of *B. bigemina*, the highest infection levels were found in bovine calves (*F* = 17.38, *p* <0.05), while the values were similar among buffalo calves, adult buffalo, and adult cattle. No correlation was found between the parasitic loads of *B. bovis* (CN/µL of DNA) in tick larvae and mammalian hosts (*r* = 0.2, *p* = 0.27). In contrast, there was a correlation between the parasite loads in tick and vertebrate hosts (*r* = 0.4, *p* = 0.03) of *B. bigemina*. There are sparse references on this subject, although Giglioti et al. [35] found a high correlation (*p* <0.01) on the infection levels of *B. bovis* (*r* = 0.58) and *B. bigemina* (*r* = 0.6) between carrier cattle and ticks. These authors suggest the existence of a cause–effect relationship between parasitic loads in cattle and ticks; additional studies should address this issue.

In general, our results showed that adult females of *R. microplus* fed on water buffalo can produce offspring infected by *B. bovis* and *B. bigemina*. This fact contributes as a piece of evidence of the participation of water buffalo as a potential reservoir of *B. bovis* and *B. bigemina*. The potential impact of these results for the epidemiology of babesiosis in western Cuba, and perhaps in other endemic regions where cattle and buffalo are bred together, remains to be fully elucidated. Further investigation is needed to assess: (1) Whether uninfected ticks can acquire *Babesia* when feeding on *Babesia*-infected buffaloes and (2) whether transovarial transmission of *Babesia* is equally efficient in uninfected ticks that acquire the pathogen while feeding on the buffalo as in ticks carrying the infection from previous generations. Another important question that remains to be answered is the relative contribution of horizontal transmission and vertical transmission in the epidemiological cycles of the system *R. microplus*-buffalo-*Babesia*.

## 3. Materials and Methods

### 3.1. Study Site and Samples’ Selection

**Figure 1:**
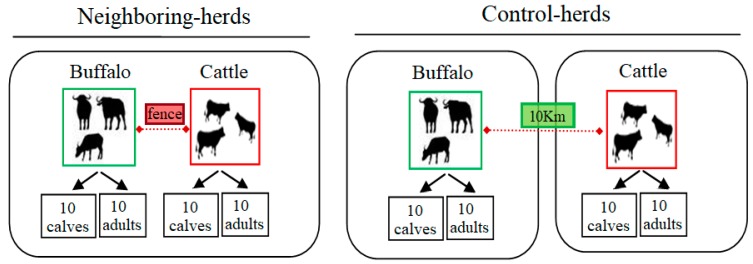
Diagram of experimental design. A cohort study was carried out, with two study groups, consisting of 20 buffaloes and 20 cattle each. In one group, bovines and buffalo cohabited on adjoining ranches, with grazing areas divided by a perimeter fence (Neighboring herds), while the other group was the reference panel (Control herds), consisting of two groups of cattle and buffalo from distant herds (~10 km between them). Besides, each subgroup consisted of 10 calves (0–1 year) and 10 adult animals (>5 years).

A cohort study was conducted in livestock of the municipality of San Jose, Mayabeque, Cuba. The climate of the region is tropical, seasonally humid, with an annual average of temperature between 22 and 28 °C, and relative humidity of 80% [37]. *R. microplus* is the only tick species identified in cattle and buffaloes of the region [38]. This tick species persists throughout the year with peaks of high-abundance population during the dry season (December–March) [38]. *B. bovis*, *B. bigemina,* and *A. marginale* are endemic in cattle populations of this region [4]. The prevalence of these pathogens in buffalo of the region is high [22,39]. For this study, two farms were selected where cattle and buffalo herds were raised together, either in immediate proximity separated by wire fences (hereafter the “Neighboring herd” group) or separated by a distance of 10 km (hereafter the “Control herd” group). Each group, neighboring and control, consisted of 20 cattle and 20 buffalo randomly selected. Each cohort consisted of 10 calves (0–1 year) and 10 adults (>5 years). The experimental design is shown in Figure 1.

### 3.2. Tick Infestation

Adult female ticks (≥4.5 mm) [40] were counted on the left side of each animal, and the resulting value was multiplied by two to determine the parasite burden. This data were used to calculate the prevalence of tick infestation (TIP) (i.e., % of infested animals) and the intensity of tick infestation (TIR) (i.e., adult females/infested animals) in each group [41]. Tick counting was done between 7:00 to 8:00 a.m. [42]. The nymph/larvae ratio was compared between buffalo and bovine calves to determine the larval survival rate. For this purpose, the tick life stages were counted as described by Veríssimo et al. [43]; briefly, a 10 cm diameter area of the perineal region of each animal was scraped with a razor blade (Figure S1). The scraping material was collected and placed in tubes with a screw cap (15 mL) containing 3 mL of distilled water. The material was transferred to Petri dishes, and up to 100 ticks were counted on each plate using a Stemi DV4 Stereo microscope (Carl Zeiss, Oberkochen, Germany). In the counts, the numbers of larvae or nymphs were discriminated, and adult ticks were not considered. The nymph/larvae ratio was calculated as: Nymphs (%) = (nymphs/nymphs + larvae) × 100.

### 3.3. Analysis of the Reproductive Parameters of Adult Female Ticks

The reproductive performance was evaluated in adult female ticks collected from 31 hosts (12 buffalo calves, nine bovine calves, and 10 adult cattle). Ticks were manually detached at the end of engorgement (≥4.5 mm, with dorsal grooves not visible, [42]) and placed in 15-mL conical tubes, covered with cotton. In the laboratory, groups of up to 10 adult females were placed in Petri dishes and incubated at 27 ± 1 °C and 85–86% relative humidity for 15 days for oviposition to occur, as described by Oliveira et al. [44]. The egg mass of each group of adult females was weighed, placed in closed syringes with cotton, and incubated for 21 days. The larval hatching rate (HR) was determined as previously described by Giglioti et al. [45]. Three estimators of the reproductive performance of female ticks were calculated: (1) Conversion efficiency (CE): CE% = (WE / WT) × 100; (2) hatching rate (HR): HR% = (NL/NL + NE) × 100; and (3) reproductive efficiency index (REI): REI = (WE × IR × 20,000)/WT, where: WE is the weight of eggs, WT is the weight of female, NL is the number of larvae, NE is the number of eggs not incubated, and 20,000 is an estimated number of larvae that are produced from 1 g of eggs.

### 3.4. DNA Extraction and Quantification of Babesia Infection Levels

A blood sample was collected from each animal in 4 mL vacutainer EDTA tubes (BD Vacutainer Blood Collection, Franklin Lakes, NJ, USA), and kept frozen at −80 °C in cryovials until processing. The genomic DNA was extracted from 300 µL of blood by using the Wizard^®^ Genomic DNA Purification Kit (Promega, Madison, WI, USA) according to the manufacturer’s instructions. For DNA extraction from ticks, approximately 100 larvae were taken from each sample, poured into a sterile 1.5 mL Eppendorf tube, flash-frozen in liquid nitrogen and immediately crushed with a plastic macerator, and homogenized in a Tissue Lyser^®^ (Qiagen, Hilden, Germany). DNA extraction was performed using DNeasy^®^ Blood and Tissue DNA Purification Kit (Qiagen, Hilden, Germany). DNA quantity and quality were evaluated using a Nanodrop Spectrophotometer 1000 v.3.5 (Thermo Fisher Scientific, Waltham, MA, USA) and stored at −20 °C. The SYBR green-based qPCR was used for high sensitivity detection of *B. bovis* and *B. bigemina*. The primers used were those described by Buling et al. [46], which amplify 88 base pairs (bp) fragments of the mitochondrial cytochrome b gene (*mt-Cytb*). The standardized thermocycling conditions, as well as the quantification procedures, were as described previously by Obregon et al. [47].

### 3.5. Statistical Analysis

The TIP was compared between groups with the Fisher Exact test and Duncan test (95% confidence interval (CI)), while the ratio nymphs/larvae and the TIR were analyzed using the ANOVA test and Duncan’s multiple range test (95% CI). The ANOVA and Duncan’s multiple range tests were also used to compare the reproductive efficiency indicators (CE, HR, and REI) between groups, as well as the infection levels of hemoparasites (measured as DNA copy number (CN), CN/µL) in the DNA samples from tick larvae and mammalian hosts. Pearson’s correlation coefficient (*r*) was used to explore the association on the infection levels of *B. bovis* and *B. bigemina* between tick larvae and the hosts wherein the adult females fed. The analyses were performed using the statistical software package Statgraphics Centurion v. 16.1.03 (StatPoint technologies Inc, Warrenton, VA, USA).

### 3.6. Ethics Statement

All procedures in this work were according to the principles established by the International Guiding Principles for Biomedical Research Involving Animals (2012). The committee on ethics and animal welfare at Centro Nacional de Sanidad Agropecuaria (CENSA) approved this research. No animal was sacrificed for the purposes of the study and the field study did not involve endangered or protected species. The epidemiological survey did not involve harm or cruelty to animals. The border veterinary service of the Institute of Veterinary Medicine (IMV) from Cuba authorized the exportation of DNA samples used in this work (certification number R.S.1522010).

## 4. Conclusions

With this investigation, we concluded that *R. microplus* infestation, although rare in adult buffaloes, is similar in frequency and intensity in cattle and buffalo calves living in endemic areas in western Cuba. We found that the reproductive performance of female ticks that fed on buffalo calves was similar to that of female ticks that fed on cattle. More importantly, the offspring of adult females fed on buffalo calves carried *B. bovis* and *B. bigemina*, suggesting transovarial transmission of *Babesia* spp. in ticks fed on buffalo calves. Furthermore, the levels of *Babesia* DNA were similar in the offspring of adult females that fed on buffalo and cattle. Future studies are needed to establish the transmission rate of *Babesia* spp. in regions where the life cycle of *R. microplus* is completed in buffaloes. Other components of the possible reservoir competence of water buffalo for *B. bovis* and *B. bigemina*, such as the infectiousness of the strains transmitted by ticks fed on buffalos, should also be analyzed.

## Figures and Tables

**Table 1:** *R. microplus* infestation on buffalo and cattle from different ages and closeness groups.

		Neighboring-Herds		Control-Herds	
Indicators *	Age Group	Cattle	Buffalo	Cattle	Buffalo
(TIP) Tick infestation prevalence	Adults	80%	0%	60%	0%
	Calves	50%	100%	70%	60%
(TIR) Tick infestation rate ($\bar{x}$ ± SD)	Adults	7 ± 3^ab^	0	5 ± 2^b^	0
	Calves	7 ± 4^ab^	12 ± 10^a^	5 ± 3^b^	5 ± 4^b^
Nymphs/larvae ratio ($\bar{x}$ ± SE)	Adults	-	-	-	-
	Calves	36 ± 0.009%^c^	30 ± 0.009%^b^	35 ± 0.009%^c^	24 ± 0.008%^a^

* Different letters in the same indicator indicate values significantly different between groups (*p* ≤ 0.05).

**Table 2:** Reproductive performance of *R. microplus* engorged females fed on buffaloes and cattle from different age groups and distances between herds.

		Neighboring-Herds		Control-Herds	
Indicators	Age Group	Cattle	Buffalo	Cattle	Buffalo
(CE) Conversion efficiency ($\bar{x}$ ± SE)	Adults	43 ± 0.04%	-	42 ± 0.07%	-
	Calves	38 ± 0.05%	49 ± 0.03%	41 ± 0.05%	50 ± 0.07%
(IR) Incubation rate ($\bar{x}$ ± SE)	Adults	98 ± 0.02%	-	99 ± 0.04%	-
	Calves	98 ± 0.02%	95 ± 0.01%	96 ± 0.03%	99 ± 0.04%
(REI) Reprod. efficiency index ($\bar{x}$ ± SD)	Adults	8.4 ± 2.0 × 10^5^	-	8.4 ± 5.2 × 10^5^	-
	Calves	7.4 ± 3.0 × 10^5^	9.5 ± 2.2 × 10^5^	7.9 ± 1.6 × 10^5^	9.8 ± 2.0 × 10^5^

**Table 3:** Molecular quantification (qPCR) of *B. bovis* and *B. bigemina* infection in the offspring of adult females of *R. microplus* fed on buffalo and cattle.

		Neighboring-Herds		Control-Herds	
Hemoparasite *	Age Group	Cattle	Buffalo	Cattle	Buffalo
*B. bovis (*_Log_CN/µL)	Adults	8.5 ± 1.3^b^	-	8.6 ± 1.4^b^	-
	Calves	7.8 ± 0.7^b^	7.3 ± 0.6^a^	7.7 ± 1.0^b^	7.2 ± 0.5^a^
*B. bigemina* (_Log_CN/µL)	Adults	5.3 ± 1.0^ab^	-	4.8 ± 1.4^a^	-
	Calves	7.2 ± 1.3^b^	5.6 ± 0.7^a^	6.0 ± 2.0^ab^	5.4 ± 0.4^a^

* Different letters in the same hemoparasite indicate values significantly different between groups (*p* ≤ 0.05).

**Table 4:** Molecular quantification (qPCR) of *B. bovis* and *B. bigemina* infection on carrier cattle and buffaloes.

		Neighboring-Herds		Control-Herds	
Hemoparasite *	Age Group	Cattle	Buffalo	Cattle	Buffalo
*B. bovis* (_Log_CN/µL)	Adults	8.1 ± 0.3^c^	6.6 ± 1.4^ab^	8.0 ± 0.0^bc^	7.5 ± 1.0^bc^
	Calves	8.3 ± 0.3^c^	6.7 ± 0.9^a^	8.1 ± 0.3^c^	7.5 ± 0.1^abc^
*B. bigemina* (_Log_CN/µL)	Adults	6.1 ± 0.8^b^	5.0 ± 1.0^ab^	6.3 ± 1.1^abc^	4.8 ± 1.8^ab^
	Calves	9.0 ± 0.7^d^	6.5 ± 0.7^ab^	8.4 ± 0.7^cd^	5.0 ± 0.1^a^

* Different letters in the same hemoparasite indicate values significantly different between groups (*p* ≤ 0.05).

## Data Availability

All data generated or analyzed during this study are included in this published article.

## Supplementary Materials

The following are available online at https://www.mdpi.com/2076-0817/9/4/280/s1, Figure S1: Tick sampling procedure for tick nymph/larval ratio analysis. The figures represent the main four stages of sampling: (1) First, a circular area (10 cm in diameter) was delimited in the perineal region of each bovine and buffalo calf, (2) then the skin was moistened with soapy water, (3) and was shaved with a razor and sterile blade, and (4) the scraping was placed in capped tubes (15 mL) containing 3 mL of distilled water. Figure S2: The skin of buffalo calves in anatomic areas infested with *R. microplus* ticks. The arrow’s point indicates lesions due to inflammatory reactions in response to bites and tick fixation. Figure S3: Real-time qPCR analysis of tick larvae for babesial hemoparasites. (**a**) Amplification signal from tick DNA samples on the qPCR assay for *B. bovis.* (**b**) Melting curve analysis of resulting *B. bovis* amplicons (melting peaks at 79.5 °C. (**c**) Amplification signal from tick DNA samples on the qPCR assay for *B. bigemina.* (**d**) Melting curve analysis of resulting *B. bigemina* amplicons (melting peaks at 78.5 °C. Each sample was analyzed in duplicate, NTC, no template control. Graphics automatically generated by the software Bio-Rad CFX96 Manager v. 3.1 (BioRad, USA).
