# Supplementary material for: Efficient Transovarial Transmission of Babesia Spp. in Rhipicephalus microplus Ticks Fed on Water Buffalo (Bubalus bubalis)
Source: Pathogens. 2020 Apr 11;9(4):280. doi: 10.3390/pathogens9040280 (PMC7238136; doi:10.3390/pathogens9040280)
Supplement: Supplementary file 1 [file pathogens-09-00280-s001.pdf]

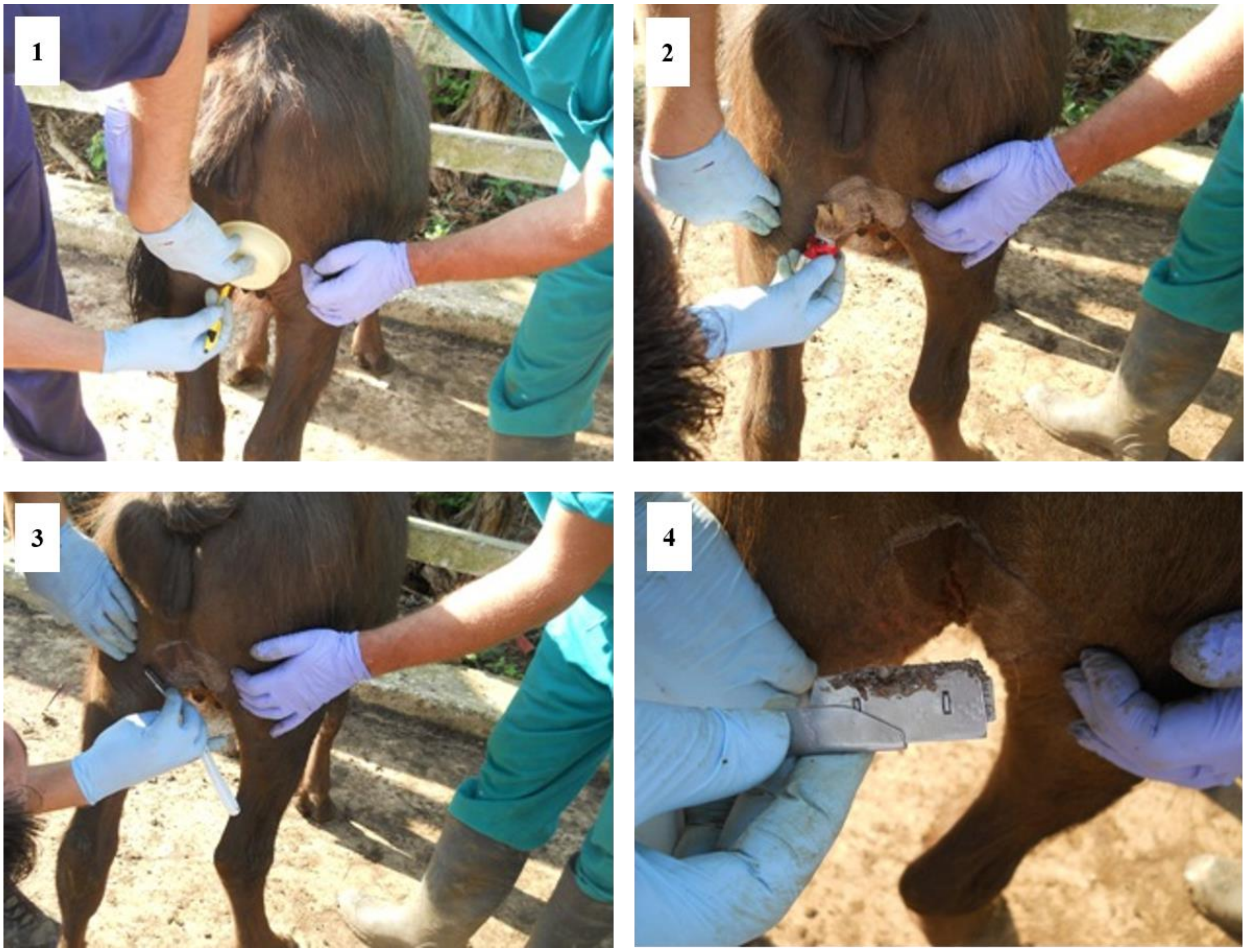

Supplementary Figure 1. Tick sampling procedure for tick nymph/larval ratio analysis. The figures represent the main four stages of sampling. 1) First, a circular area (10 cm in diameter) was delimited in the perineal region of each bovine and buffalo calf, 2) then the skin was moistened with soapy water, 3) and was shaved with a razor and sterile blade, 4) the scraping was placed in caps tubes (15mL) containing 3 mL of distilled water.

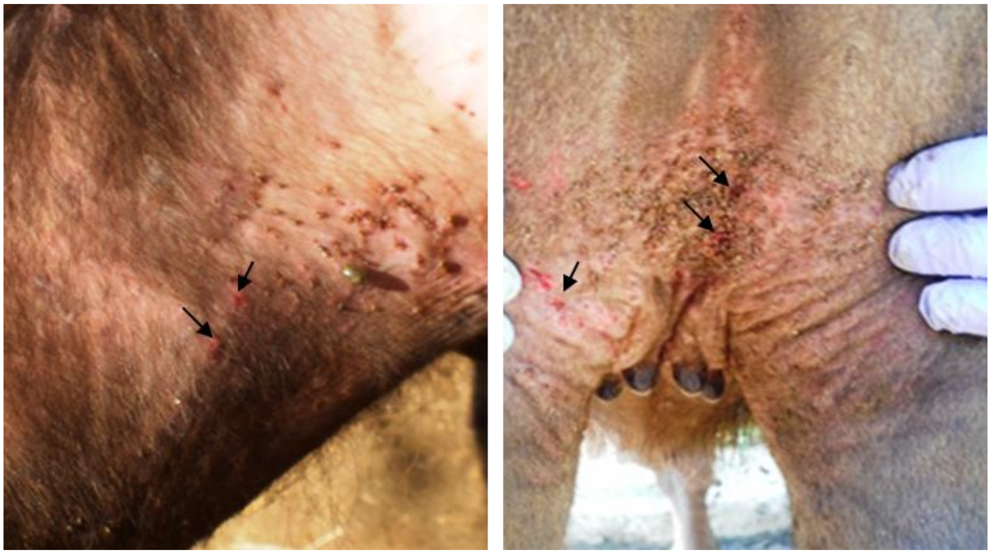

Supplementary Figure 2. The skin of buffalo calves in anatomic areas infested with *R. microplus* ticks.

The arrows point indicates lesions due to inflammatory reactions in response to bites and tick fixation.

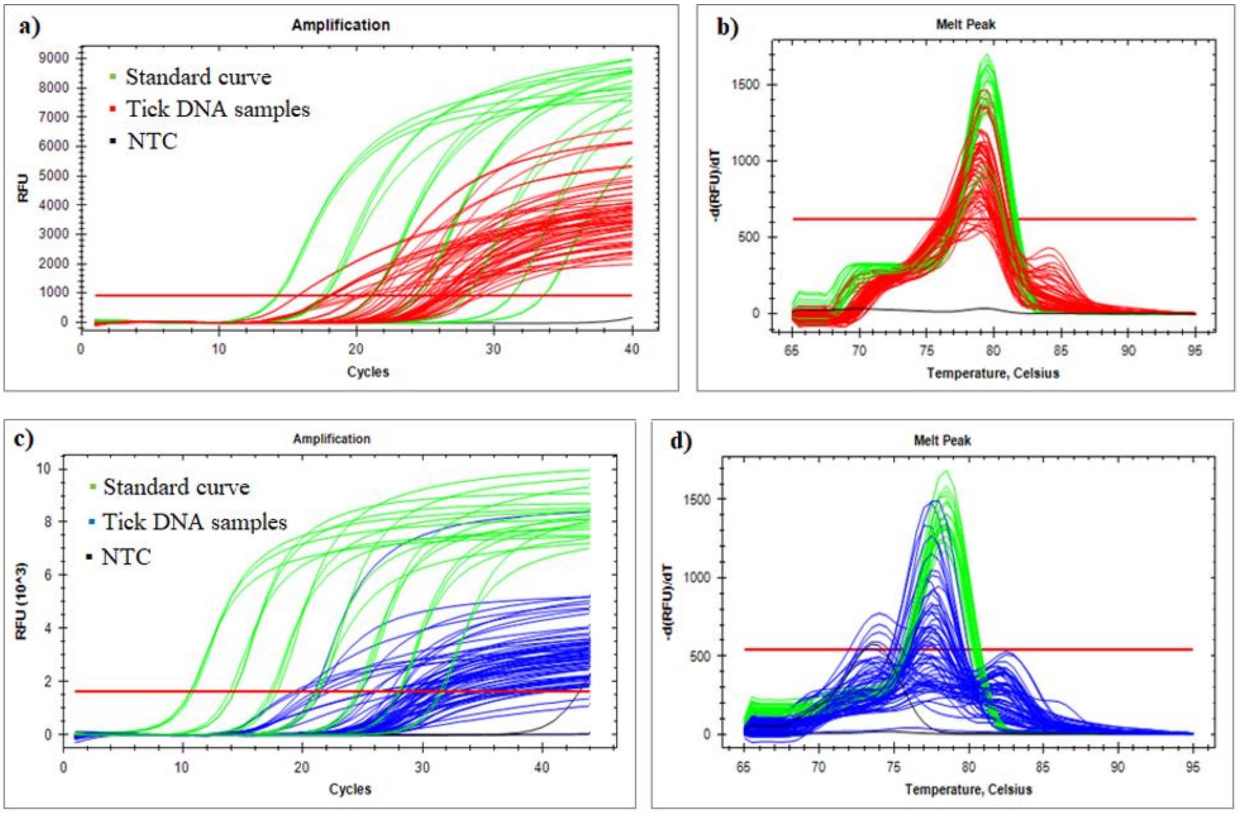

Supplementary Figure 3. Real-time qPCR analysis of tick larvae for babesial hemoparasites. **a)** Amplification signal from tick DNA samples on the qPCR assay for *B. bovis.* **b)** Melting curve analysis of resulting *B. bovis* amplicons (melting peaks at 79.5ºC. **c)** Amplification signal from tick DNA samples on the qPCR assay for *B. bigemina.* **d)** Melting curve analysis of resulting *B. bigemina* amplicons (melting peaks at 78.5ºC. Each sample was analyzed in duplicate, NTC- no template control. Graphics automatically generated by the software Bio-Rad CFX96 Manager v. 3.1 (BioRad, USA).
